# Proportion of neonatal sepsis and determinant factors among neonates admitted in University of Gondar comprehensive specialized hospital neonatal Intensive care unit Northwest Ethiopia 2017

**DOI:** 10.1186/s13104-019-4587-3

**Published:** 2019-08-27

**Authors:** Ayenew Engida Yismaw, Tirngo Yihunie Abebil, Mulunesh Abuhay Biweta, Bilen Mekonnen Araya

**Affiliations:** 1 College of Medicine and Health Science, School of Midwifery, University of Gondar, Gondar, Ethiopia; 2 Amhara Regional State Health Bureau Merawi Primary Hospital, Merawi, Ethiopia

**Keywords:** Neonatal sepsis, Premature rupture of membrane, Neonatal intensive care unit, Gondar

## Abstract

**Objective:**

Neonatal sepsis is one of the most common causes of neonatal hospitalization in developing countries. It is also a major cause of mortality in the world affecting both developed and developing countries. Diagnosis and management of sepsis are a great challenge facing neonatologists in neonatal intensive care units due to nonspecific signs and symptoms. This study, therefore, was aimed to determine proportion and risk factors of neonatal sepsis at university of Gondar comprehensive specialized hospital, North West Ethiopia.

**Result:**

The proportion of neonatal sepsis was 11.7%. Factors significantly associated with neonatal sepsis were: Neonatal related factors were: Premature rupture of membrane (AOR = 2.74; 95% Cl (1.39, 5.38), congenital anomaly (AOR = 3.14; 95% CI (1.09, 10.28), and low Apgar score (AOR = 2.69; 95% Cl (1.37, 5.26). Maternal factors were: foul-smelling vaginal discharge (AOR = 2.75; 95% Cl (1.40, 5.38), and Intrapartum fever (AOR = 3.35; 95% Cl (1.7, 6.62). In this finding proportion of Neonatal sepsis was low as compared to previous studies. Measures targeting the prevention of premature rupture of membranes and low Apgar score need to be taken, like strengthening maternal birth preparedness and complication readiness plans. Also, identification of congenital anomalies earlier in pregnancy and taking measures to avoid birth injury may decrease neonatal sepsis.

## Introduction

Neonatal sepsis is defined as a clinical syndrome characterized by systemic signs and symptom of an inflammatory response in the presence of or as a result of suspected or proven infection during the first month of life [1]. It is a major cause of morbidity and mortality worldwide accounting about 26% [2]. Especially, neonates in low-income countries where identification and specific treatment of the bacteria are often unsatisfactory were more affected. It is possible to save most cases of neonatal sepsis by using timely gold standard diagnosis and sensitive antibiotics treatment with good supportive care [3].

Neonatal infections are most prevalent and high case fatality risk associated with severe bacterial infections which accounts 9.8% were found in the low-income countries of sub-Saharan Africa, South Asia, and Latin America [4]. The routes of transmission were not much known globally where different environmental risk factors may affect paths of transmission. However, Neonatal sepsis mostly affects low-birth-weight infants, neonates who undergo invasive procedures during their hospitalization in the Neonatal Intensive Care Unit (NICU) and also acquired horizontally from the environment or vertically from mother [5].

Around half of neonatal deaths caused by sepsis occur during the first week of life and remains a feared and life-threating complication, especially among very low birth weight (VLBW) and preterm infants [6].

Many researchers have studied the common causative agents of neonatal sepsis with their sensitivity patterns. However, limited studies tried to verify the risk factors of neonatal sepsis in the study area as well as in the country as a whole. This study, therefore, was aimed to determine proportion and risk factors of neonatal sepsis at university of Gondar comprehensive specialized hospital, North West Ethiopia.

## Main text

### Methods

Institution-based cross-sectional study design was employed at University of Gondar Comprehensive specialized hospital neonatal intensive care unit from September 1 to November 30, 2017, which is located in Amhara national regional state, northwest Ethiopia. The hospital is one of the largest teaching hospitals found in Amhara region providing tertiary level care for more than seven million people in the North West part of the country coming from Amhara, Tigray, and Benishangul Gumuz regions. The NICU provides an outpatient and inpatient medical service for neonates. Neonatal intensive care unit particularly offers intensive care for neonates in inpatient basis. Source population were all neonates admitted in neonatal intensive care unit at University of Gondar comprehensive specialized hospital and study population were all neonates admitted in neonatal intensive care unit at University of Gondar comprehensive specialized hospital during the study period. From all neonates admitted in neonatal intensive care unit blood sample was taken and culture has been done. Based on the results reported those neonates with blood culture positive were classified as has neonatal sepsis and those neonates with blood culture-negative were classified as has no neonatal sepsis.

The sample size was determined by using single population proportion formula with the assumption of 95% level of confidence, 5% marginal error, and by taking 44.7% prevalence [7]. By considering 10% non-response rate, the final sample size was 423. Systematic random sampling technique was employed to get study units from neonates admitted at neonatal intensive care unit. Sampling interval (K) was calculated by taking the 3 months neonates’ admission report from NICU which was 1350. Then K = N/n, 1350/423 = 3.19 ≈ 3. Every 3rd woman with her neonate was interviewed, the first woman was selected randomly.

Data were collected by interviewer-administered questionnaire adapted with modification from different pieces of literatures. The questionnaire was first prepared in English, translated to Amharic and then translated back to English to check conceptual consistency. A pre-test was done on 5% of the samples at neonatal intensive care unit other than the study site. Supportive supervision was carried out during the entire data collection period.

Data were cleaned, and entered into Epi-info version 7 and transferred to SPSS version 20 for analysis. First descriptive statistics were applied and presented using texts, tables and graphs of variables and followed by bivariate and multivariable logistic regression analysis to test statistical association. Variables showed statistical association in the bivariate logistic analysis with *P* value < 0.25 were entered to multivariable logistic regression. The strength of association was interpreted using adjusted odds ratio and confidence interval with P-value < 0.05.

### Result

A total of 423 neonates admitted in neonatal intensive care unit with their index mothers were included in the study, making the response rate 100%. The median age of mothers was 25 ± 5.8 years, ranging from 18 to 45 years (Table 1).

**Table 1:** Socio demographic characteristics of mothers with their index neonates admitted in University of Gondar comprehensive specialized hospital neonatal intensive care unit North West Ethiopia, 2017 (n = 423)

Variables	Categories	Frequency (n = 411)	Percent (%)
Age of mothers	< 35 years	260	61.5
	≥ 35 years	163	38.5
Residence	Urban	327	77.3
	Rural	96	22.7
Marital status	Currently married	346	81.4
	Currently un married	77	18.6
Religion	Orthodox	384	90.8
	Muslim	35	8.3
	Protestant	4	0.9
Educational level	Not attended formal education	315	74.5
	Attended formal education	108	25.5
Occupation	House wife	135	31.9
	Employed	179	42.3
	Others^a^	109	25.8
Sex of neonate	Male	221	52.5
	Female	202	47.5
Age of neonate	1–7 days	417	98.1
	8–28 days	6	1.9

Others^a^ = students, daily laborers and merchants

The median (± IQ range) of parity for cases was 2 ± 1 which ranges from 1 to 8 live births. Most of the mothers delivered at health institution by SVD (Table 2).

**Table 2:** Obstetric characteristics of respondents with their index neonate who were admitted in University of Gondar comprehensive specialized hospital neonatal intensive care unit Northwest Ethiopia, 2017 (n = 423)

Variables	Categories	Frequency (n = 411)	Percent (%)
Parity	Primipara	248	58.6
	Multi para	175	41.4
PPROM	Yes	121	28.6
	No	302	71.4
APH	Yes	27	6.4
	No	396	93.6
ANC follow up	Yes	380	89.83
	No	43	10.17
Place of delivery	Health institution	405	95.7
	Home	18	4.3
Mode of delivery	SVD	340	80.3
	C/S	46	10.9
	Instrumental	37	8.7
Duration of labor	< 24 h	377	89.1
	≥ 24 h	46	10.9

Health related risk factors among mothers were 40% UTI, 26% foul-smelling vaginal discharge, 16% febrile illness, 15% chronic illness and 3% had STI.

Out of the 423 neonates 47(11.1%) with (95% CI 8.2, 14.4) had positive blood culture for sepsis.

Thirty-one point six percent of neonates were resuscitated at birth, 74% of neonates were breastfeed exclusively and 22.2% of neonates received formula feeding.

About 41.8% neonates were preterm, 31.6% had low Apgar score (less than 3), 13.7% had low birthweight, 4.5% had congenital anomaly and 3.8% had prolonged hospital stay.

In bivariate analysis parity, foul-smelling vaginal discharge, PPROM, intrapartum fever, congenital anomaly and Apgar score were associated with the outcome variable. Of those five of them were statistically significant in multivariable analysis.

In multivariable analysis prolonged premature rupture of membrane (AOR = 2.74; 95% CI (1.39, 5.38), congenital anomaly (AOR = 3.14; 95% CI (1.09, 10.28), foul-smelling vaginal discharge(AOR = 2.75; 95% CI (1.40, 5.38),low Apgar score (AOR = 2.69; 95% CI (1.37, 5.26) and Intrapartum fever (AOR = 3.35; 95% CI (1.7, 6.62) were significantly associated with neonatal sepsis (Table 3).

**Table 3:** Proportion and associated factors of neonatal sepsis among neonates admitted in University of Gondar comprehensive specialized Hospital Neonatal Intensive Care Unit, Northwest Ethiopia, 2017 (n = 423)

Variables	Neonatal sepsis		COR (95% cl)	AOR (95% cl)
	Yes	No		
Parity				
Multi para	26	149	1.88 (1.02,3.47)	1.009 (0.23, 2.09)
Primi para	21	227	1	
Intrapartum fever				
Yes	24	86	3.51 (1.89,6.54)	3.35 (1.70, 6.62)**
No	23	290	1	1
Foul smelling Vaginal discharge				
Yes	27	104	3.53 (1.89,6.54)	2.75 (1.40, 5.38)*
No	20	272	1	1
PROM				
Yes	25	96	3.31 (1.78,6.14)	2.74 (1.39, 5.38)*
No	22	280	1	1
Apgar score				
Low Apgar	26	108	3.07 (1.65,5.69)	2.69 (1.37, 5.26)*
Normal Apgar	21	268	1	1
Congenital anomaly				
Yes	6	13	4.43 (1.58,12.45)	3.14 (1.09, 10.28)*
No	40	364	1	1

** P-value < 0.001, * P-value < 0.05

### Discussion

This study presented that, proportions of culture proven neonatal sepsis among neonates admitted at NICU was 11.1% with (95% CI (8.2, 14.4)). Which was in line with the study done in Nepal which was 12.6% [8]. The result was greater than the study done in Mexico which was 4.3% [9]. This might be due to difference in study setting and health system set up.

However, it was lower than studies done in Iran (52.4%), India (63.4%), Nigeria (43.5%), Brazil (68.1%), Egypt (38.5%), Singapore (24.6%), Addis Ababa (44.7%) and Gondar (32.04%) [7, 10–16]. It might be because of differences in study period and study setup.

Neonates who had low Apgar score at birth were 2.7 times at risk of having neonatal sepsis compared to neonates with good Apgar score at birth AOR = 2.69(1.37, 5.26). This was consistent with studies done in Mexico and Mekelle [9, 17]. Low APGAR score leads to an immunological insult and resuscitation. The resuscitation procedures following birth asphyxia exposes neonates to sepsis causative microbes. Neonates born to mothers having PPROM > 12 h had a 2.7 times risk of having neonatal sepsis compared to those neonates born before 12 h of PROM (AOR = 2.74 (95%Cl 1.39, 5.38)). This finding was similar to studies conducted in Mexico [9], Brazil [16] and Mekelle [17]. Early and prolonged rupture of membrane increases the risk of ascending infection from the birth canal into the amniotic sac and fetal membrane leading to asphyxia, which frequently causes sepsis.

Neonates born from mothers who had foul smelling Vaginal discharge had 2.7 times odds of developing sepsis compared to neonates born from mothers who did not have foul smelling Vaginal discharge (AOR = 2.75(95% Cl (1.4, 5.38)). This might be due to that, foul smelling vaginal discharge is indicative of vaginal colonization with group B streptococcus (GBS) which leads to ascending infection from organisms that colonize the perineum and genitourinary (GU) tract of the mother.

Neonates born from mothers’ who had fever during labor had 3.3 times risk of developing sepsis compared to their counterparts (AOR = 3.35 95% Cl (1.7, 6.62)). This is similar with the study done in Mekelle [17]. The fact that fever is a sign of infection which can be transmitted to the neonate leading to sepsis.

Neonates who had congenital anomaly were 3.1 times at risk of having neonatal sepsis from those who didn’t have congenital anomaly AOR = 3.14 95% Cl (1.09, 10.28). This might be due to that open spinal bifida, which was found on the neonates is suitable for entrance and multiplication of microorganisms. On the other hand, neonates who had congenital anomaly had a chance of longer period of hospital admission which exposes the neonate to hospital acquired neonatal sepsis.

### Conclusion

Proportions of neonatal sepsis in this study was low compared to previous studies. History of foul-smelling vaginal discharge, PROM, low Apgar score at 5th minute, intrapartum fever and congenital anomaly were found to be associated factors of neonatal sepsis. Measures targeting the prevention of premature rupture of membranes and low Apgar score need to be taken by the government and none governmental organizations to improve quality of health care and health care facilities to avoid preventable risk factors like foul-smelling discharge, febrile illness, PROM, and low APGAR. Also, identification of congenital anomalies earlier in pregnancy and taking preventive measures of birth injury may decrease neonatal sepsis.

## Limitation

It was a cross sectional study and may not show clear cause and effect relationship. And also the finding was based on blood culture only which may not definitive diagnostic method and not address on identifying causative agents.

## Data Availability

Data will be obtained up on request by emailing to the corresponding author using “ayenewe07@gmail.com”.

## Abbreviations

C/S: Cesarean Section
CONS: Coagulase-Negative Staphylococcus
EOS: early onset sepsis
GBS: Group B Staphylococcus
LOS: late onset sepsis
LBW: low birth weight
NICU: Neonatal Intensive Care Unit
NI: nosocomial infection
PROM: premature rupture of membrane
STD: sexually transmitted disease
SVD: spontaneous vaginal delivery
TBA: traditional birth attendant
UTI: urinary tract infection
VLBW: very low birth weight
